# 2-[(*E*)-3-Phenyl­prop-2-en­yl]-1,2-benzisothia­zol-3(2*H*)-one 1,1-dioxide

**DOI:** 10.1107/S1600536809012999

**Published:** 2009-04-08

**Authors:** Muhammad Nadeem Arshad, Hafiz Mubashar-ur-Rehman, Muhammad Zia-ur-Rehman, Islam Ullah Khan, Muhammad Shafique

**Affiliations:** a Department of Chemistry, Government College University, Lahore 54000, Pakistan; b Applied Chemistry Research Centre, PCSIR Laboratories Complex, Ferozpure Road, Lahore 54600, Pakistan

## Abstract

In the crystal structure of the title compound, C_16_H_13_NO_3_S, the benzisothia­zole group is almost planar (r.m.s. deviation for all non-H atoms excluding the two O atoms bonded to S = 0.009 Å). The dihedral angle between the fused ring and the terminal ring is 13.8 (1)°. In the crystal, mol­ecules are linked through inter­molecular C—H⋯O contacts forming a chain of mol­ecules along *b*.

## Related literature

For the synthesis of benzothia­zine and benzisothia­zol derivatives, see: Zia-ur-Rehman *et al.* (2006 ▶, 2009 ▶); Siddiqui *et al.* (2008 ▶). For the biological activity of benzisothia­zols, see: Kapui *et al.* (2003 ▶); Liang *et al.* (2006 ▶). For related structures, see: Siddiqui *et al.* (2006 ▶, 2007*a* ▶,*b* ▶,*c* ▶).
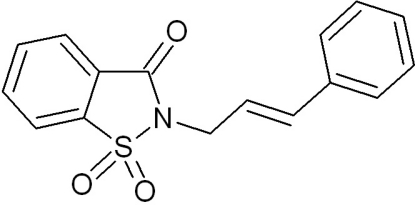


## Experimental

### 

#### Crystal data

C_16_H_13_NO_3_S
*M* *_r_* = 299.33
Monoclinic, 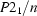

*a* = 6.9375 (5) Å
*b* = 7.1579 (4) Å
*c* = 29.673 (2) Å
β = 96.160 (4)°
*V* = 1464.99 (17) Å^3^
*Z* = 4
Mo *K*α radiation
μ = 0.23 mm^−1^
*T* = 296 K
0.39 × 0.11 × 0.10 mm

#### Data collection

Bruker APEXII CCD area-detector diffractometer
Absorption correction: none
8250 measured reflections
3606 independent reflections
1722 reflections with *I* > 2σ(*I*)
*R*_int_ = 0.034

#### Refinement

*R*[*F* ^2^ > 2σ(*F* ^2^)] = 0.051
*wR*(*F* ^2^) = 0.178
*S* = 0.96
3606 reflections
190 parameters
H-atom parameters constrained
Δρ_max_ = 0.32 e Å^−3^
Δρ_min_ = −0.40 e Å^−3^

### 

Data collection: *APEX2* (Bruker, 2007 ▶); cell refinement: *SMART* (Bruker, 2007 ▶); data reduction: *SAINT*; program(s) used to solve structure: *SHELXS97* (Sheldrick, 2008 ▶); program(s) used to refine structure: *SHELXL97* (Sheldrick, 2008 ▶); molecular graphics: *PLATON* (Spek, 2009 ▶) and *Mercury* (Macrae *et al.*, 2006 ▶); software used to prepare material for publication: *SHELXTL* (Sheldrick, 2008 ▶) and local programs.

## Supplementary Material

Crystal structure: contains datablocks I, global. DOI: 10.1107/S1600536809012999/bt2924sup1.cif

Structure factors: contains datablocks I. DOI: 10.1107/S1600536809012999/bt2924Isup2.hkl

Additional supplementary materials:  crystallographic information; 3D view; checkCIF report

## Figures and Tables

**Table 1:** Hydrogen-bond geometry (Å, °)

*D*—H⋯*A*	*D*—H	H⋯*A*	*D*⋯*A*	*D*—H⋯*A*
C2—H2⋯O1^i^	0.93	2.29	3.174 (4)	158

Symmetry code: (i) 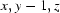.

**Fig. 1.**
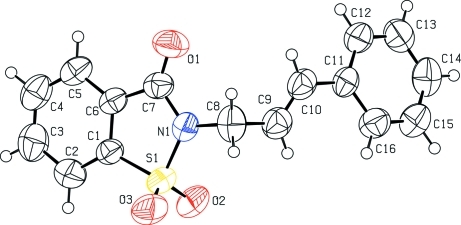
The asymmetric unit of the title compound. Displacement ellipsoids are drawn at the 50% probability level.

**Fig. 2.**
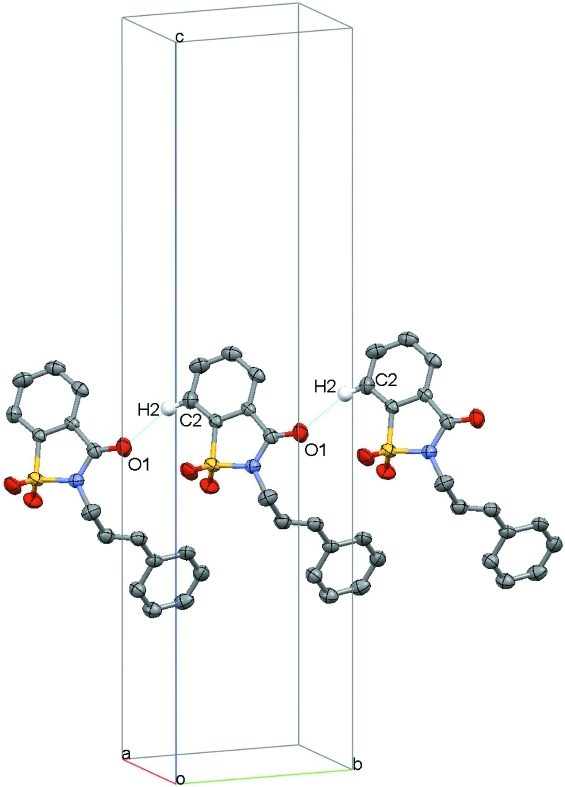
Perspective view of the crystal packing showing inter molecular C—H···O interactions (dashed lines). H atoms not involved in hydrogen bonding have been omitted for clarity.

## supplementary crystallographic information

### Comment

Benzisothiazolone-1,1-dioxide and its various derivatives are well known as biologically active compounds *e.g.*, saccharin has been identified as an important molecular component in various classes of 5-HTla antagonists, analgesics and human mast cell tryptase inhibitors (Liang *et al.*, 2006). Few of its derivatives are considered to be the most potent orally active human leucocyte elastase (HLE) inhibitors for the treatment ofchronic obstructive pulmonary disease (COPD), acute respiratory distress syndrome (ARDS), cystic fibrosis, asthma and other inflammatory diseases (Kapui *et al.*, 2003). Its *N*-alkyl derivatives have been successfully transformed to non-steroidal anti-inflammatory drugs *e.g.*, piroxicam (Zia-ur-Rehman *et al.*, 2006).

In continuation to our research on the synthesis of 1,2-benzothiazine 1,1-dioxide derivatives (Zia-ur-Rehman *et al.*, 2009; Zia-ur-Rehman *et al.*, 2006), we have in addtion, worked on the synthesis of benzisothiazole derivatives (Siddiqui *et al.*, 2006; Siddiqui *et al.*, 2008). Herein, crystal structure of the title compound (I) is described. The benzisothiazole moiety is exactly planar.The molecular dimensions are in accord with the corresponding dimensions reported in similar structures (Siddiqui *et al.*, 2007*a*-c). Each molecule is linked to its adjacent one through C—H···O contacts forming a chain of molecules along *b*.

### Experimental

A mixture of 2,3-dihydro-1,2-benzisothiazol-3-one-1,1-dioxide (1.83 g, 10.0 mmoles), dimethyl formamide (5.0 ml) and cinnamyl chloride (1.67 g, 10.0 mmoles) was stirred for a period of three hours at 90°C. Contents were cooled to room temperature; poured over crushed ice to get white coloured precipitates which were filtered, washed and dried. Crystallization of the white precipitates (in methanol) afforded suitable crystals for X-ray studies after recrystalization in methanol.

### Refinement

H atoms bound to C were placed in geometric positions (C—H distance = 0.93 to 0.96 Å) using a riding model with *U*_iso_(H) = 1.2 *U*_eq_(C) or *U*_iso_(H) = 1.5 *U*_eq_(C _methyl_).

### Crystal data

**Table:**

C_16_H_13_NO_3_S	*F*(000) = 624
*M**_r_* = 299.33	*D*_x_ = 1.357 Mg m^−^^3^
Monoclinic, *P*2_1_/*n*	Mo *K*α radiation, λ = 0.71073 Å
Hall symbol: -P 2yn	Cell parameters from 1453 reflections
*a* = 6.9375 (5) Å	θ = 2.8–20.7°
*b* = 7.1579 (4) Å	µ = 0.23 mm^−^^1^
*c* = 29.673 (2) Å	*T* = 296 K
β = 96.160 (4)°	Needles, white
*V* = 1464.99 (17) Å^3^	0.39 × 0.11 × 0.10 mm
*Z* = 4	

### Data collection

**Table:**

Bruker APEXII CCD area-detector diffractometer	1722 reflections with *I* > 2σ(*I*)
Radiation source: fine-focus sealed tube	*R*_int_ = 0.034
graphite	θ_max_ = 28.3°, θ_min_ = 1.4°
φ and ω scans	*h* = −9→8
8250 measured reflections	*k* = −8→9
3606 independent reflections	*l* = −35→39

### Refinement

**Table:**

Refinement on *F*^2^	Primary atom site location: structure-invariant direct methods
Least-squares matrix: full	Secondary atom site location: difference Fourier map
*R*[*F*^2^ > 2σ(*F*^2^)] = 0.051	Hydrogen site location: inferred from neighbouring sites
*wR*(*F*^2^) = 0.178	H-atom parameters constrained
*S* = 0.96	*w* = 1/[σ^2^(*F*_o_^2^) + (0.0875*P*)^2^] where *P* = (*F*_o_^2^ + 2*F*_c_^2^)/3
3606 reflections	(Δ/σ)_max_ < 0.001
190 parameters	Δρ_max_ = 0.32 e Å^−^^3^
0 restraints	Δρ_min_ = −0.40 e Å^−^^3^

### Special details

**Table:**

Geometry. All e.s.d.'s (except the e.s.d. in the dihedral angle between two l.s. planes) are estimated using the full covariance matrix. The cell e.s.d.'s are taken into account individually in the estimation of e.s.d.'s in distances, angles and torsion angles; correlations between e.s.d.'s in cell parameters are only used when they are defined by crystal symmetry. An approximate (isotropic) treatment of cell e.s.d.'s is used for estimating e.s.d.'s involving l.s. planes.
Refinement. Refinement of *F*^2^ against ALL reflections. The weighted *R*-factor *wR* and goodness of fit *S* are based on *F*^2^, conventional *R*-factors *R* are based on *F*, with *F* set to zero for negative *F*^2^. The threshold expression of *F*^2^ > σ(*F*^2^) is used only for calculating *R*-factors(gt) *etc*. and is not relevant to the choice of reflections for refinement. *R*-factors based on *F*^2^ are statistically about twice as large as those based on *F*, and *R*- factors based on ALL data will be even larger.

### Fractional atomic coordinates and isotropic or equivalent isotropic displacement parameters (Å^2^)

**Table:**

	*x*	*y*	*z*	*U*_iso_*/*U*_eq_	
S1	0.25184 (11)	−0.21860 (10)	0.08353 (3)	0.0673 (3)	
O1	0.2457 (3)	0.2827 (3)	0.05026 (8)	0.0871 (7)	
O2	0.4314 (3)	−0.2892 (3)	0.10446 (8)	0.0937 (8)	
O3	0.0787 (3)	−0.2970 (3)	0.09666 (7)	0.0909 (7)	
N1	0.2464 (3)	0.0118 (3)	0.09024 (7)	0.0634 (6)	
C1	0.2500 (4)	−0.1992 (4)	0.02512 (9)	0.0552 (7)	
C2	0.2505 (4)	−0.3412 (4)	−0.00625 (12)	0.0800 (9)	
H2	0.2512	−0.4661	0.0025	0.096*	
C3	0.2499 (5)	−0.2902 (6)	−0.05133 (12)	0.0914 (11)	
H3	0.2495	−0.3827	−0.0733	0.110*	
C4	0.2498 (4)	−0.1074 (6)	−0.06412 (11)	0.0805 (9)	
H4	0.2496	−0.0775	−0.0946	0.097*	
C5	0.2501 (4)	0.0333 (5)	−0.03269 (10)	0.0654 (8)	
H5	0.2500	0.1578	−0.0416	0.078*	
C6	0.2506 (3)	−0.0140 (4)	0.01241 (8)	0.0532 (6)	
C7	0.2488 (4)	0.1141 (4)	0.05129 (10)	0.0609 (7)	
C8	0.2380 (4)	0.0994 (5)	0.13459 (10)	0.0799 (9)	
H8A	0.1591	0.0230	0.1524	0.096*	
H8B	0.1762	0.2206	0.1304	0.096*	
C9	0.4368 (4)	0.1238 (5)	0.16027 (10)	0.0735 (8)	
H9	0.4949	0.0193	0.1746	0.088*	
C10	0.5312 (5)	0.2786 (4)	0.16377 (9)	0.0700 (8)	
H10	0.4704	0.3826	0.1499	0.084*	
C11	0.7266 (4)	0.3074 (4)	0.18770 (9)	0.0603 (7)	
C12	0.8373 (5)	0.4574 (4)	0.17645 (9)	0.0768 (9)	
H12	0.7871	0.5416	0.1544	0.092*	
C13	1.0228 (5)	0.4829 (5)	0.19796 (11)	0.0834 (10)	
H13	1.0982	0.5821	0.1897	0.100*	
C14	1.0949 (5)	0.3626 (5)	0.23127 (12)	0.0838 (10)	
H14	1.2193	0.3801	0.2457	0.101*	
C15	0.9854 (5)	0.2174 (5)	0.24335 (11)	0.0799 (9)	
H15	1.0337	0.1375	0.2666	0.096*	
C16	0.8052 (5)	0.1884 (4)	0.22158 (10)	0.0745 (9)	
H16	0.7332	0.0865	0.2296	0.089*	

### Atomic displacement parameters (Å^2^)

**Table:**

	*U*^11^	*U*^22^	*U*^33^	*U*^12^	*U*^13^	*U*^23^
S1	0.0771 (6)	0.0647 (5)	0.0584 (5)	0.0005 (4)	−0.0004 (4)	0.0158 (4)
O1	0.1019 (17)	0.0544 (13)	0.1044 (19)	0.0035 (11)	0.0083 (13)	0.0060 (11)
O2	0.1041 (17)	0.0869 (15)	0.0828 (16)	0.0187 (12)	−0.0234 (13)	0.0209 (12)
O3	0.1006 (17)	0.0970 (17)	0.0785 (16)	−0.0211 (12)	0.0253 (13)	0.0253 (12)
N1	0.0710 (15)	0.0670 (15)	0.0509 (14)	0.0007 (11)	0.0009 (11)	0.0008 (11)
C1	0.0510 (15)	0.0568 (16)	0.0570 (16)	−0.0012 (11)	0.0015 (13)	0.0099 (13)
C2	0.098 (2)	0.0619 (19)	0.080 (2)	−0.0007 (16)	0.0071 (19)	−0.0011 (17)
C3	0.104 (3)	0.107 (3)	0.064 (2)	0.004 (2)	0.013 (2)	−0.014 (2)
C4	0.067 (2)	0.113 (3)	0.062 (2)	0.0019 (18)	0.0115 (16)	0.012 (2)
C5	0.0494 (16)	0.081 (2)	0.0669 (19)	0.0027 (14)	0.0093 (14)	0.0217 (17)
C6	0.0398 (14)	0.0625 (17)	0.0572 (16)	0.0007 (11)	0.0043 (12)	0.0131 (13)
C7	0.0500 (16)	0.0592 (19)	0.073 (2)	0.0009 (12)	0.0023 (14)	0.0112 (15)
C8	0.072 (2)	0.100 (2)	0.068 (2)	−0.0014 (17)	0.0060 (16)	−0.0131 (17)
C9	0.082 (2)	0.079 (2)	0.0603 (19)	0.0036 (17)	0.0112 (16)	0.0005 (15)
C10	0.084 (2)	0.074 (2)	0.0532 (18)	0.0113 (17)	0.0096 (16)	0.0003 (14)
C11	0.0657 (18)	0.0722 (19)	0.0438 (15)	0.0036 (14)	0.0103 (14)	−0.0014 (13)
C12	0.106 (3)	0.076 (2)	0.0497 (17)	−0.0063 (18)	0.0141 (17)	0.0012 (15)
C13	0.100 (3)	0.089 (2)	0.065 (2)	−0.0285 (19)	0.0236 (19)	−0.0086 (18)
C14	0.067 (2)	0.115 (3)	0.069 (2)	−0.0069 (19)	0.0076 (17)	−0.012 (2)
C15	0.073 (2)	0.095 (2)	0.070 (2)	0.0056 (18)	0.0020 (18)	0.0096 (18)
C16	0.073 (2)	0.078 (2)	0.072 (2)	−0.0025 (15)	0.0068 (17)	0.0110 (16)

### Geometric parameters (Å, °)

**Table:**

S1—O3	1.418 (2)	C8—C9	1.512 (4)
S1—O2	1.424 (2)	C8—H8A	0.9700
S1—N1	1.662 (2)	C8—H8B	0.9700
S1—C1	1.738 (3)	C9—C10	1.286 (4)
O1—C7	1.207 (3)	C9—H9	0.9300
N1—C7	1.370 (3)	C10—C11	1.476 (4)
N1—C8	1.464 (3)	C10—H10	0.9300
C1—C6	1.378 (3)	C11—C12	1.382 (4)
C1—C2	1.378 (4)	C11—C16	1.384 (4)
C2—C3	1.387 (4)	C12—C13	1.386 (4)
C2—H2	0.9300	C12—H12	0.9300
C3—C4	1.362 (5)	C13—C14	1.365 (4)
C3—H3	0.9300	C13—H13	0.9300
C4—C5	1.372 (4)	C14—C15	1.358 (4)
C4—H4	0.9300	C14—H14	0.9300
C5—C6	1.380 (3)	C15—C16	1.360 (4)
C5—H5	0.9300	C15—H15	0.9300
C6—C7	1.475 (4)	C16—H16	0.9300
			
O3—S1—O2	117.84 (14)	N1—C8—C9	112.4 (2)
O3—S1—N1	109.21 (13)	N1—C8—H8A	109.1
O2—S1—N1	109.29 (12)	C9—C8—H8A	109.1
O3—S1—C1	112.95 (13)	N1—C8—H8B	109.1
O2—S1—C1	112.08 (14)	C9—C8—H8B	109.1
N1—S1—C1	92.43 (12)	H8A—C8—H8B	107.9
C7—N1—C8	122.3 (3)	C10—C9—C8	124.7 (3)
C7—N1—S1	115.28 (19)	C10—C9—H9	117.7
C8—N1—S1	122.4 (2)	C8—C9—H9	117.7
C6—C1—C2	121.6 (3)	C9—C10—C11	126.3 (3)
C6—C1—S1	110.5 (2)	C9—C10—H10	116.8
C2—C1—S1	127.9 (2)	C11—C10—H10	116.8
C1—C2—C3	117.2 (3)	C12—C11—C16	117.9 (3)
C1—C2—H2	121.4	C12—C11—C10	119.8 (3)
C3—C2—H2	121.4	C16—C11—C10	122.3 (3)
C4—C3—C2	121.5 (3)	C11—C12—C13	120.3 (3)
C4—C3—H3	119.3	C11—C12—H12	119.9
C2—C3—H3	119.3	C13—C12—H12	119.9
C3—C4—C5	121.0 (3)	C14—C13—C12	120.0 (3)
C3—C4—H4	119.5	C14—C13—H13	120.0
C5—C4—H4	119.5	C12—C13—H13	120.0
C4—C5—C6	118.6 (3)	C15—C14—C13	120.1 (3)
C4—C5—H5	120.7	C15—C14—H14	119.9
C6—C5—H5	120.7	C13—C14—H14	119.9
C1—C6—C5	120.1 (3)	C14—C15—C16	120.2 (3)
C1—C6—C7	112.6 (2)	C14—C15—H15	119.9
C5—C6—C7	127.3 (3)	C16—C15—H15	119.9
O1—C7—N1	123.6 (3)	C15—C16—C11	121.5 (3)
O1—C7—C6	127.1 (3)	C15—C16—H16	119.3
N1—C7—C6	109.2 (2)	C11—C16—H16	119.3
			
O3—S1—N1—C7	−117.2 (2)	C8—N1—C7—O1	0.6 (4)
O2—S1—N1—C7	112.6 (2)	S1—N1—C7—O1	−179.9 (2)
C1—S1—N1—C7	−1.9 (2)	C8—N1—C7—C6	−178.0 (2)
O3—S1—N1—C8	62.3 (2)	S1—N1—C7—C6	1.5 (3)
O2—S1—N1—C8	−67.9 (2)	C1—C6—C7—O1	−178.7 (3)
C1—S1—N1—C8	177.7 (2)	C5—C6—C7—O1	0.4 (4)
O3—S1—C1—C6	113.7 (2)	C1—C6—C7—N1	−0.2 (3)
O2—S1—C1—C6	−110.3 (2)	C5—C6—C7—N1	178.9 (2)
N1—S1—C1—C6	1.6 (2)	C7—N1—C8—C9	−94.9 (3)
O3—S1—C1—C2	−67.1 (3)	S1—N1—C8—C9	85.6 (3)
O2—S1—C1—C2	68.9 (3)	N1—C8—C9—C10	101.9 (4)
N1—S1—C1—C2	−179.2 (3)	C8—C9—C10—C11	−178.7 (3)
C6—C1—C2—C3	−0.5 (4)	C9—C10—C11—C12	157.8 (3)
S1—C1—C2—C3	−179.6 (2)	C9—C10—C11—C16	−22.3 (5)
C1—C2—C3—C4	0.3 (5)	C16—C11—C12—C13	1.6 (4)
C2—C3—C4—C5	−0.1 (5)	C10—C11—C12—C13	−178.4 (3)
C3—C4—C5—C6	0.0 (4)	C11—C12—C13—C14	−1.8 (5)
C2—C1—C6—C5	0.5 (4)	C12—C13—C14—C15	0.1 (5)
S1—C1—C6—C5	179.73 (19)	C13—C14—C15—C16	1.6 (5)
C2—C1—C6—C7	179.7 (2)	C14—C15—C16—C11	−1.8 (5)
S1—C1—C6—C7	−1.1 (3)	C12—C11—C16—C15	0.1 (4)
C4—C5—C6—C1	−0.2 (4)	C10—C11—C16—C15	−179.8 (3)
C4—C5—C6—C7	−179.3 (2)		

### Hydrogen-bond geometry (Å, °)

**Table:**

*D*—H···*A*	*D*—H	H···*A*	*D*···*A*	*D*—H···*A*
C2—H2···O1^i^	0.93	2.29	3.174 (4)	158

Symmetry codes: (i) *x*, *y*−1, *z*.
